# Cultural Beliefs About Diabetes-Related Social Exclusion and Diabetes Distress Impact Self-Care Behaviors and HbA1c Among Patients with Type 2 Diabetes

**DOI:** 10.1007/s12529-023-10179-w

**Published:** 2023-05-30

**Authors:** Albert L. Ly, Patricia M. Flynn, Hector M. Betancourt

**Affiliations:** 1 Department of Psychology, Loma Linda University, Loma Linda, CA 92350 USA; 2 Department of Preventive Medicine, Loma Linda University, Loma Linda, CA 92350 USA

**Keywords:** Cultural beliefs, Diabetes distress, Diabetes self-care, HbA1c, Type 2 diabetes, Latinos

## Abstract

**Background:**

Type 2 diabetes mellitus (T2DM) disproportionally impacts Latin Americans (Latinos) in the U.S. compared to non-Latino Whites, as reflected by an increased risk for disease complications and higher mortality rates. Guided by an Integrative Model of Culture, Psychological Processes, and Health Behavior, the purpose of the present study was to examine the role of cultural beliefs and diabetes distress as determinants of self-care behaviors and HbA1c among Latino patients with T2DM.

**Methods:**

Participants included 109 Latino patients with T2DM recruited from a diabetes treatment center located in a region of Southern California with high diabetes mortality rates. Structural equation modeling was employed to examine the extent to which cultural beliefs about diabetes-related social exclusion and diabetes distress impact self-care behaviors and self-reported HbA1c.

**Results:**

Consistent with the study hypotheses, cultural beliefs about diabetes-related social exclusion predicted diabetes distress, which in turn predicted poor diabetes self-care.

**Conclusions:**

Findings suggest an important need for intervention efforts that address both cultural and psychological factors in order to improve diabetes self-care behaviors and associated disease outcomes among Latino patients with T2DM. Future research could benefit from investigating protective aspects of culture that could help counter the negative implications of cultural beliefs about social exclusion and diabetes distress associated with poor self-care.

## Introduction

Type 2 diabetes mellitus (T2DM) is a global epidemic which disproportionately impacts racial/ethnic minority populations and socioeconomically disadvantaged communities. For example, Latin Americans in the United States (Latinos)^1^ are approximately 1.7 times more likely to have T2DM as compared to non-Latino Whites (NLW; [2]). Moreover, Latinos are more likely to develop complications from diabetes than NLWs as evidenced by a 1.5 times greater risk for visual impairment, 2.0 times greater risk for end stage renal disease, and 1.3 times greater likelihood of death [2]. The treatment of T2DM involves engaging in a variety of diabetes self-care behaviors across multiple domains including glucose monitoring, dietary intake, physical activity, medication adherence, and regular eye and foot exams [3]. Research reveals that T2DM treatment adherence is worse among Latinos as compared to NLW particularly in the domains of medication, blood glucose monitoring, and self-foot exams (for a review see [4]). Poor self-care leads to higher levels of HbA1c as well as a greater risk for developing serious complications [5–8]. Hence, there is a critical need to better understand the cultural and psychological factors relevant to diabetes self-care behaviors among Latino patients in the United States in order to reduce the deleterious outcomes related to this chronic disease.

Research reveals that the complex and demanding treatment regimens associated with T2DM are often appraised negatively by patients resulting in increased distress [9, 10]. Distress that manifests from the burden of living with diabetes [11], or *diabetes distress*, broadly refers to patient concerns about diabetes management, support, the emotional burden of the disease, and access to care [12]. Findings from an international study of diabetes patients from 17 countries, including the U.S. and Mexico, revealed that up to 45% of patients with T2DM experience diabetes-related distress [13]. This experience of diabetes distress is also more prevalent among racial/ethnic minority patients as compared to non-Latino White patients in the U.S. [14–16]. Moreover, one study revealed higher rates of diabetes distress among Latino (*M* = 33) as compared to African American (*M* = 19) patients [17]. Further, several studies have demonstrated the negative impact of diabetes distress on self-care behaviors and glycemic control among diverse patient populations in the U.S., including African Americans and non-Latino Whites [18], as well as among Latinos in the U.S. [19] and in South America [20].

Mitigating the complications of T2DM and related disparities among Latino patients in the U.S. requires the development of culturally responsive interventions that can effectively improve the management of this chronic disease. While culture is increasingly recognized as an important factor relevant to the management of chronic diseases (e.g., [21, 22]), considerably less is known concerning how and to what extent these cultural beliefs, values, norms, and practices impact psychological factors such as diabetes distress, self-care, and subsequent disease outcomes. One notable factor that has been found to be socially shared among diabetes patients in Latin America pertains to beliefs about social exclusion [20]. Using a mixed-methods cultural research approach [23], qualitative interviews were conducted with diabetes patients in Latin America resulting in the identification of socially shared beliefs concerning exclusion, rejection, and discrimination due to diabetes. These socially shared beliefs about social exclusion were found to predict higher treatment distress and worse glycemic control among patients with T2DM [20].

**Fig. 1:**
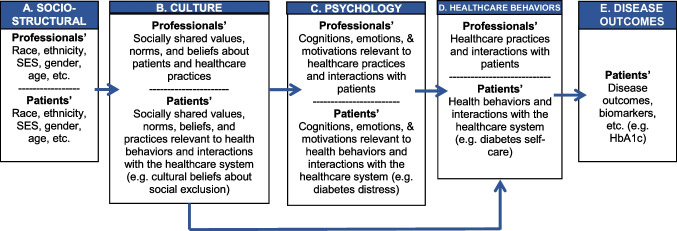
Betancourt's Integrative Model of Culture, Psychology, and Behavior: Adapted to Account for Behaviors and Outcomes Relevant to Type 2 Diabetes Mellitus

While research examining *cultural beliefs* about diabetes-related social exclusion [20] shares some similarities with constructs such as stigma, discrimination, and social rejection, these constructs are conceptually distinct. Namely, the latter constructs have traditionally been investigated from the perspective of an *individual’s perception or experience of* stigma, discrimination, social exclusion, and social rejection, while the former reflects *cultural or socially shared beliefs* that diabetes patients are rejected, discriminated, and excluded. The bottom-up cultural research approach [23] is a methodology that has reliably identified *socially shared* (i.e. cultural) beliefs in a particular population or cultural group (see [20, 24, 25]). As previously discussed, prior research employing this cultural research approach identified beliefs about diabetes-related social exclusion that were socially shared (e.g. cultural) among T2DM patients of Latin American backgrounds [20]. According to this operationalization of culture, when a Latino patient endorses an item such as, “a patient with diabetes feels excluded because of their disease”, it does not necessarly mean that this particular patient personally experienced exclusion. Rather, it indicates that this individual, much like other Latino T2DM patients, endorses a commonly held cultural belief that T2DM patients are excluded due to their disease. According to the Integrative Model of Culture and Behavior guiding this research (see Fig. 1), beliefs that are *cultural or socially shared* among members of a particular community (e.g., patients with T2DM) have the potential to impact psychological processes (e.g., diabetes distress) and related behaviors (e.g., diabetes self-care) and outcomes (e.g., HbA1c).

The broader literature on *perceived or experienced* social exclusion, discrimination, and stigma may provide preliminary evidence for the need to further explore the cultural construct among T2DM patients in the U.S. Social exclusion has been broadly defined as the experience of being kept apart from others emotionally (e.g., ignored) or physically (e.g., isolated) through rejection or ostracism [26]. The core experience of rejection, which involves being told or implied that one is not wanted, can also include experiences of discrimination and stigmatization. One can feel excluded in interpersonal relationships as well as at the societal level through messaging that one’s social group is devalued (e.g. stigmatization) [27]. Hence, there is some conceptual overlap between the experience of social exclusion at an interpersonal level due to one’s diabetes diagnosis and the experience of diabetes-related stigma and discrimination at the societal level. One of the contributions of this paper is that individuals who believe that diabetes patients are excluded or discriminated can be negatively impacted by this cultural belief, even though they may not have personally experienced exclusion or discrimination due to their diabetes.

Research investigating the perception or experience of discrimination among T2DM patients reveals significant associations with diabetes distress, poor diabetes care, HbA1c, and disease complications [28–31]. Less research has been conducted in the U.S. on diabetes stigma. However, one exploratory study (*N* = 53) revealed that over half of participants perceived stigma in their family [32]. Another study including predominantly non-Latino White T2DM patients revealed that 52% perceived diabetes stigma, which was associated with greater HbA1c [33]. It should be noted that much of the research on perceived or experienced discrimination and stigma conducted in the U.S. included samples of participants from various racial/ethnic groups or predominantly non-Latino White patients. From a cultural perspective, a more nuanced understanding of the impact of these constructs appears necessary, particularly among specific racial/ethnic populations. Such an approach is especially needed to better understand the experience of T2DM among Latinos in the U.S., who experience high rates of this disease compared to non-Latino Whites, and are historically underrepresented in medical research.

Given the higher rates of diabetes distress and poor self-care among Latino patients as compared to NLWs, and in some cases African Americans [4, 14, 15, 17], there is a critical need for research to systematically examine how cultural beliefs associated with T2DM relate to psychological distress, and in turn impact diabetes self-care and outcome. While multiple determinants of diabetes self-care have been identified in the literature (e.g., social structural, psychological, cultural), less is known regarding the multivariate nature of relations among these variables. Experts argue that in order to better understand the role of social and psychological determinants of health behaviors, integrative theoretical frameworks are needed [34]. As such, the use of integrative models and multivariate statistical approaches could significantly advance knowledge in this area and lead to the development of more effective, culturally responsive interventions [24, 34].

Betancourt’s Integrative Model of Culture, Psychological Processes, and Health Behavior [23, 24, 35] is one such model that specifies the structure of relations between social structural, cultural, and psychological factors as determinants of health behaviors such as those involved in the management of T2DM (see Fig. 1). According to the Integrative Model (see Fig. 1), aspects of culture (B), such as the socially shared beliefs﻿, values, norms, and practices relevant to T2DM, can directly impact health behavior (D), including self-care behaviors involved in the management of T2DM (e.g., adhering to the diabetes diet, exercise, and medication regimens). These aspects of culture can also indirectly influence health behavior through psychological processes (C), such as the experience of distress associated with diabetes and medical treatment. Aspects of culture (B) may be socially shared among members of groups, such as those based on race, ethnicity, SES, gender, country of birth, and other identities or communities (A). Health behaviors can also translate into biological outcomes (E), such as HbA1c in the context of T2DM.

This integrative model has been used to examine the role of culture in cancer screening disparities [24, 36, 37], patient-professional relations [25], seeking healthcare services [38], healthcare mistreatment [39], and diabetes outcomes [20]. Collectively, these studies confirm the proposed structure of relations postulated by the Integrative Model. Namely, several of these studies revealed that the impact of culture on the corresponding health behaviors and outcomes was indirect, through various mediating psychological processes [20, 25, 36, 39]. And in some cases, there was both a direct, as well as an indirect effect of culture on health behavior and outcome [20, 38].

## The Present Study

Guided by Betancourt’s Integrative Model of Culture, the aim of the present study was to systematically examine the complex nature of relations among cultural beliefs about diabetes-related social exclusion and diabetes distress as determinants of self-care and HbA1c among Latino patients with T2DM in Southern California. The research was conducted in San Bernardino County, California, a community where Latinos comprise the majority population (54.4%; [41]). San Bernardino County also has some of the highest rates of T2DM in the state (13.9% versus state average of 9.9%) and the 3^rd^ highest diabetes death rates out of 58 California counties [41].

Consistent with the Integrative Model, it was hypothesized that cultural beliefs about diabetes-related social exclusion would directly and/or indirectly impact diabetes self-care through diabetes distress, and that diabetes self-care would, in turn, predict HbA1c. Specifically, it was hypothesized that: 1) higher scores on cultural beliefs about diabetes-related social exclusion (B in Fig. 1) would predict higher scores on diabetes distress (C); 2) higher scores on diabetes distress (C) would predict poor diabetes self-care (D), and; 3) poor diabetes self-care (D) would be associated with higher HbA1c (E).

## Method

### Participants

A total of 109 Latino patients with T2DM were recruited from a diabetes treatment center in San Bernardino County in Southern California. Criteria for inclusion were having a diagnosis of T2DM, being at least 18 years of age, self-identifying as Latino American, and able to read English or Spanish. One case was found to be an outlier, resulting in a final sample of 108. The mean age of participants was 55.74 (*SD* = 12.27), 63.9% identified as women, and the average years of education attained was 12.25 (*SD* = 3.07). Additional demographic information is provided in Table 1.

**Table 1:** Descriptive Statistics for Participant Sample (N = 108)

	*n* (%)
Age, mean (*SD*)	55.74 (12.27)
Gender	
Female	69 (63.9%)
Male	28 (25.9%)
Household Income	
< $14,999	11 (10.2%)
$15,000–24,999	13 (12.0%)
$25,000–39,999	24 (22.2%)
$40,000–59,999	14 (13.0%)
$60,000–79,999	12 (11.1%)
$80,000–100,000	13 (12.0%)
> $100,000	9 (8.3%)
Education (in years), mean (*SD*)	12.25 (3.07)
Ethnicity	
Latin American	108 (100.0%)
Mexican	38 (35.2%)
Puerto Rican	7 (6.5%)
Central American	3 (2.8%)
South American	1 (0.9%)
Other	5 (4.6%)
Did not specify	54 (50.0%)
Country of Birth	
United States	63 (58.3%)
Outside the U.S.	45 (41.7%)
Years diagnosed with T2DM, mean (*SD*)	10.57 (10.73)

## Procedures

Approval for the study was obtained from the Institutional Review Board at the authors’ university prior to data collection. Participants were recruited from diabetes education classes at a diabetes treatment center with permission from site administrators. Data collection occurred during the second of four diabetes education classes. At the start of the scheduled class time, bilingual Spanish–English research assistants provided a summary of the study and described eligibility criteria, risks and benefits of participation, and the estimated time to complete the instrument (10–15 min). Interested participants provided informed consent and were administered a paper-and-pencil instrument in either English or Spanish. Participants were provided the opportunity to be entered into a drawing for a $50 Amazon gift card as an incentive for participating in the study.

## Measures

### Social Structural Sources of Cultural Variation

Participants responded to items assessing age, gender, yearly household income, education, country of birth, and length of time since diagnosis. According to the Integrative Model [23], these social-structural variables are considered potential sources of variation in cultural beliefs. Participants indicated their yearly household income in U.S. dollars based on five income categories, ranging from “less than $15,000” to “more than $100,000.” Education level was reported in total years obtained, from one year to 20 years or greater. Participants also indicated their ethnic background (e.g., Latino American) and noted their specific ethnic heritage (e.g., Mexican, Central American, Puerto Rican). Country of birth was reported as a free-response item and then transformed into a dichotomous variable for the current study (born in the U.S., born outside of the U.S.). Participants indicated the length of time since their diagnosis in years and months.

### Cultural Beliefs about Diabetes-Related Social Exclusion

The Cultural Beliefs about Diabetes-Related Social Exclusion Scale was developed utilizing the bottom-up cultural research approach to instrument development [23], with T2DM patients in Latin America. This cultural research approach begins with specific observations relevant to an area of research (e.g. treatment adherence), which are derived through interviews with the population of interest (e.g. Latino diabetes patients). To this end, qualitative interviews with 50 T2DM patients were conducted to identify *socially shared* beliefs, values, norms, and practices relevant to T2DM and treatment adherence. Observations that were *socially shared* among the diabetes patients, were identified as cultural in nature, and quantitative items were then developed to assess these cultural beliefs, values, norms, and practices. An advantage of this approach is that it allows for the identification of aspects of culture directly from the population of interest, rather than based on stereotypical views.

The scale that resulted from this bottom-up cultural research approach was then psychometrically validated with a sample of culturally diverse patients with T2DM in Southern California [42, 43]. This three-item cultural beliefs scale assesses socially shared beliefs relevant to exclusion, rejection, and discrimination due to diabetes. Participants were asked to think about patients who have diabetes and indicate the extent to which they agreed with the following statements, “a person with diabetes feels excluded because of their disease,” “a person with diabetes feels rejected for doing the things recommended to control the disease,” and “a person with diabetes feels discriminated against because of their disease.” Item responses were based on a Likert scale ranging from 1 (*not at all true*) to 7 (*very true*). While participants provided individual scores for each of these items, the items were previously identified as *cultural or socially shared* through the bottom-up cultural research approach. Hence, higher scores indicated greater endorsement of this cultural belief by the participant responding to the cultural scale. This scale demonstrated good reliability in the present study (α = .84).

### Diabetes Distress

The Diabetes Distress Scale-2 (DDS-2; [12]), an abbreviated version of the DDS-17 [44], includes two items that measure the level of distress patients experience in the management of their diabetes. The DDS-2 is a brief and psychometrically robust screening instrument with strong reliability as demonstrated in previous research [12]. This scale has been utilized in prior research to examine diabetes distress in relation to various social structural factors (e.g., gender, ethnicity), psychological factors (e.g., chronic stress, negative life events), diabetes-related health behaviors (e.g., diet and exercise), and T2DM outcomes (e.g., diabetic complications, HbA1c; [45]). Participants rated the degree to which they felt like they were “overwhelmed by the demands of managing diabetes,” and “often failing with their diabetes regimen.” Item responses were based on a Likert scale ranging from 1 (*no distress*) to 6 (*serious distress*), with higher scores reflecting greater diabetes distress. The DDS-2 demonstrated good reliability in the present study (α = .79).

### Poor Diabetes Self-Care

The Diabetes Self-Management Questionnaire (DSMQ; [46]) includes items that measure self-care behaviors (e.g., adhering to a diabetes diet, exercise, taking medication) relevant to T2DM management. Previous research has demonstrated that the DSMQ is associated with clinical diabetes outcomes, including HbA1c [46–48]. The scaling of items was adapted and utilized in the present study to assess self-care behaviors over the past seven days. The global self-care item from the DSMQ was selected for the present research because this single item has consistently performed well across various studies [49, 50] including our own pilot study conducted with a culturally diverse sample of T2DM patients [43]. Further, a psychometric analysis of this global self-care item revealed that it has high item-total-correlations and, compared to other items in the DSMQ, was the most predictive of HbA1c [46]. Hence, the item, “How many of the past seven days has your diabetes self-care been poor?” was employed to provide an overall assessment of patients’ diabetes self-care over the past seven days. Response options ranged from 0 to 7 days. Higher scores reflect poorer diabetes self-care.

### HbA1c

HbA1c, or glycated hemoglobin, is a serologic marker that is principally used to measure diabetes treatment adherence over the past 90 days [51]. Higher levels of HbA1c reflect worse diabetes control. A HbA1c test was administered by the diabetes treatment center, which the participants reported at the time of data collection.

## Statistical Analysis

Structural equation modeling (SEM) with Maximum Likelihood (ML) estimation was used to test the study hypotheses using EQS 6.4 [52]. SEM is a particularly flexible statistical technique that can be used with many research designs, including cross-sectional data [53]. Furthermore, the analysis of mediation or indirect effects are fundamental to many SEM analyses [53]. Since SEM requires that the pattern of intervariable relations be specified a priori [54], research utilizing SEM should be guided by strong theoretical frameworks. The present study is guided by an Integrative Model of Culture and Behavior that specifies the structure of relations among socio-structural, cultural, psychological, and behavioral factors (see Fig. 1).

Data were screened for statistical assumptions, which revealed  a violation of normality. No cases with substantial missing data greater than 30% were detected. One outlier was identified and removed from the dataset prior to analysis, resulting in a final sample of 108 participants. Data were assessed for Missing Completely at Random (MCAR) via Little’s χ^2^ test of MCAR and Missing Variable Analysis in SPSS [55], which suggested that the data appeared to be missing at random. Full Information Maximum Likelihood (FIML) estimation techniques were used to handle additional cases of missing data [56].

A two-step SEM building procedure was implemented to first test the measurement model and then the full structural model [57]. Adequacy of model fit for the measurement and structural models were assessed using multiple statistical criteria suggested by Kline [58]. Robust ML estimation was employed to offset any observed non-normality of the data by providing adjusted standard errors and indices of model fit [52]. In addition, the Yuan-Bentler scaled test statistic was employed, which demonstrates robust ability to detect good model fit with small sample sizes [59]. The following criteria were used to assess good model fit: non-significant Yuan-Bentler scaled χ^2^ goodness-of-fit statistic, with a χ^2^/*df* ratio of less than 2.0; a non-robust Comparative Fit Index (CFI) of .95 or greater; a non-robust Standardized Root Mean Square Residual (SRMR) of less than .08; and a robust Root Mean Square Error of Approximation (RMSEA) of less than .08, with 90% CIs less than .10 at the upper bound [60–62]. In conjunction with theoretical and conceptual reasoning, the LaGrange and Wald post-hoc test statistics were reviewed to determine whether structural paths in the model should be added or eliminated, and if so, they were implemented in a stepwise manner [62]. To test the indirect effect of cultural beliefs on poor diabetes self-care via diabetes distress, bias-corrected bootstrapped confidence intervals (CI) were calculated using the product of coefficients method [63, 64].

## Statistical Power

**Fig. 2:**
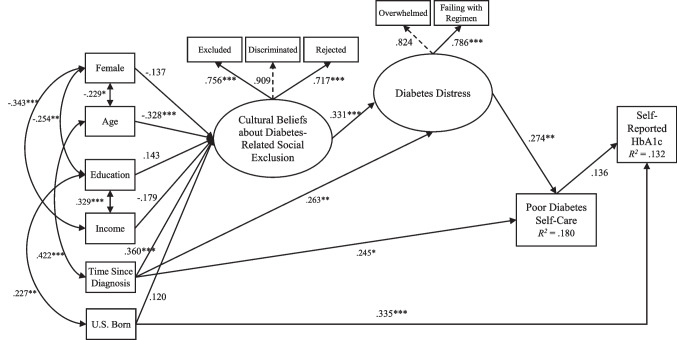
Structural equation model examining cultural beliefs and diabetes distress as predictors of poor diabetes self-care and self-reported HbA1c. Dashed lines indicate fixed paths; *p < .05, **p < .01, ***p < .001

Current methods to calculate statistical power in SEM are not well-established [65]. Multiple factors influence the power of SEM, including the associations between indicators (i.e., observations) and related constructs (i.e., latent variables), the number of latent variables, sample size, effect size, desired type 1 error rate, normality, and degrees of freedom. In general, the greater degrees of freedom a model has, the smaller sample size needed to attain the desired power level. Based on the number of latent factors and indicators for each factor, as well as the number of other indicators in the proposed model (see Fig. 2), it was estimated that a sample size of 110 participants would be necessary to achieve an 80% probability of detecting a truly significant effect [65].

## Results

### Preliminary Analyses

Table 2 reflects the means, standard deviations, and correlations among  the study variables. A review of bivariate correlations revealed several significant associations between cultural beliefs about social exclusion, diabetes distress, poor diabetes self-care, and HbA1c. The measurement model, which consisted of two latent factors (cultural beliefs about social exclusion, diabetes distress) with five indicators, fit the data well: Yuan-Bentler scaled χ^2^(4, *n* = 108) = 3.031, *p* = .552, χ^2^/*df* = .758, robust CFI = 1.000, SRMR = .020, robust RMSEA = .000, 90% CI [.000, .128]. Hence, no adjustments were made to the measurement model prior to testing the study hypotheses.

**Table 2:** Correlations, Means (*M*), and Standard Deviations (*SD*) for Study Variables

	1	2	3	4	5	6	7	8	9	10	11	12	13
1. Gender (Female)	–												
2. Age	-.265*	–											
3. Income	-.351***	-.074	–										
4. Education	-.283**	.045	.333**	–									
5. Time Since Diagnosis	-.036	.412	-.154	-.020	–								
6. Country of Birth (U.S.)	.010	-.059	.012	.223*	.072	–							
7. Social Exclusion 1	.015	-.077	-.160	.157	.137	.138	–						
8. Social Exclusion 2	-.033	-.108	-.067	.105	.220*	.158	.693***	–					
9. Social Exclusion 3	-.126	-.005	-.089	-.011	.214*	.157	.534***	.667***	–				
10. Diabetes Distress 1	.064	-.032	-.074	.004	.265*	.125	.280**	.311**	.214*	–			
11. Diabetes Distress 2	.168	-.003	-.192*	-.173	.342**	.090	.228*	.243*	.273**	.651***	–		
12. Poor Diabetes Self-Care	.066	.099	-.267**	-.096	.344**	.064	.160	.221*	.112	.304**	.274**	–	
13. Self-reported HbA1c	.064	-.098	.027	-.069	.231*	.343**	-.053	.036	-.018	.129	.228*	.165	–
*M*	–	55.74	3.55	12.25	10.57	.58	2.41	2.10	2.01	3.21	3.48	2.61	8.10
*SD*	–	12.27	1.92	3.07	10.73	.50	1.87	1.74	1.64	1.65	1.68	2.24	1.71

*p < .05; **p < .01; ***p < .001

## Tests of Study Hypotheses

﻿A structural equation model including the hypothesized theory-based relations among cultural beliefs about diabetes-related social exclusion, diabetes distress, poor self-care, and HbA1c was tested. Gender, age, household income, education, time since diagnosis, and country of birth were also included in the tested model as sources of variation in the cultural factor. A review of the fit indices and the LaGrange Multiplier test statistic suggested that adding a path from country of birth (U.S. vs. foreign born) to HbA1c, and time since diagnosis to diabetes distress, and poor diabetes self-care, would improve model fit. A review of the Wald test statistic suggested dropping the path from cultural beliefs about social exclusion to poor diabetes self-care would improve model fit. The resulting structural equation model including the hypothesized theory-based relations fit the data well: Yuan-Bentler scaled χ^2^(55, *n* = 108) = 59.253, *p* = .323, χ^2^/*df* = 1.021, CFI = .990, SRMR = .063, robust RMSEA = .027, 90% CI [.000, .067] (see Fig. 2). The study variables accounted for 18% of the variance in diabetes self-care (*R*^2^ = .180) and 13.2% of the variance in HbA1c (*R*^2^ = .132).

Consistent with the study hypotheses, cultural beliefs about social exclusion predicted diabetes distress, and diabetes distress predicted poor diabetes self-care. Specifically, higher scores on cultural beliefs about social exclusion predicted higher scores on diabetes distress (*β* = .331, *p* < .001), which in turn predicted poorer diabetes self-care (*β* = .274, *p* < .01). There was a trend in that poor diabetes self-care was associated with higher levels of HbA1c (*β* = .136, *p* > .05). Although not central to the study hypotheses, results revealed that country of birth was a significant predictor of HbA1c, such that patients born in the U.S. reported higher levels of HbA1c (*β* = .335, *p* < .001). Regarding the sources of cultural variation, greater length of time since T2DM diagnosis was significantly associated with cultural beliefs about social exclusion (*β* = .360, *p* < .001), as did younger age (*β* = -.328, *p* < .001).

## Discussion

The present study examined the role of cultural beliefs and diabetes distress as determinants of diabetes self-care and HbA1c among Latino patients with T2DM in the U.S. Consistent with the structure of relations in Betancourt’s Integrative Model of Culture, Psychology, and Health Behavior [23, 24, 35], results revealed that when participants held cultural beliefs that diabetes patients are socially excluded, rejected, or discriminated due to their disease, they were more likely to experience distress associated with managing their diabetes. Furthermore, patients who had heightened levels of diabetes distress were more likely to engage in poor diabetes self-care, which was associated with worse glycemic control. These findings provide a comprehensive understanding of the multiple determinants, namely the social-structural, cultural, and psychological factors, predictive of diabetes self-care and outcome among Latino patients with T2DM.

Findings from the present study extend prior research on diabetes distress that has largely investigated its impact on behaviors associated with diabetes self-care and outcome [20, 45, 66]. Other researchers have argued that while there is considerable research reporting higher rates of distress in ethnic minority patients with T2DM, few studies have directly investigated why this may be the case [14]. Hence, this study adds to the body of literature on diabetes distress by demonstrating that there are important cultural antecedents of diabetes distress that should also be taken into consideration. Such an approach provides greater clarity and understanding regarding the factors that contribute to increased diabetes distress, while also providing valuable information that can be used in the development of interventions to address distress.

While culture has been noted as an important factor in the context of health, the scarcity of psychometrically validated cultural instruments serves as a barrier to moving research forward in this area. The present study utilized a cultural research approach to instrument development [23] to identify cultural beliefs relevant to behaviors associated with diabetes self-care. A cultural factor that emerged from prior research conducted in Latin America had to do with patients’ beliefs that they are socially excluded due to their disease [20]. Based on results from the present study, it appears that these same cultural beliefs about social exclusion are particularly relevant to Latino patients in the U.S. Furthermore, among U.S. Latinos, an important finding was that these cultural beliefs about social exclusion led to heightened levels of diabetes distress. These findings suggest a potential benefit for interventions that address beliefs about social exclusion among diabetes patients. 

Results also highlight the various social structural factors that serve as sources of cultural variation. Specifically, patients who were younger endorsed higher levels of the socially shared beliefs about social exclusion. It may be that younger as compared to older individuals, are more impacted by cultural concerns around social exclusion. Additionally, individuals who had T2DM for a longer period of time reported higher levels of these cultural beliefs. As patients live longer with T2DM, it is possible that they are interacting with other diabetes patients to a greater extent and may have more opportunities to either experience or observe others being socially rejected or discriminated due to their T2DM. In fact, recent research suggests that individuals who lived with diabetes for 11–15 years had higher scores on a self-stigma scale compared to those that lived with T2DM less than five years [67]. Although having a family history of T2DM was not assessed in the present study, it should be taken into consideration in future research. For example, would having family members with T2DM attenuate the likelihood that an individual endorses cultural beliefs about social exclusion, or could it exacerbate the likelihood? Scollan-Koliopoulos and colleagues [68] found that patients who had family members experience social consequences due to having diabetes were more likely to have similar perceptions.

An interesting finding concerning the social structural factors included in this study was that Latino participants born in the U.S. had higher levels of HbA1c as compared to foreign-born Latinos. This is consistent with previous research demonstrating that Latinos born outside of the U.S. have better health outcomes as compared to Latinos born in the U.S. [69], and that Latino immigrants in the U.S. experience better diabetes outcomes compared to U.S. born Latinos [70]. As detailed in previous research on health outcomes among Latino immigrants in the U.S. (e.g., the Latino Paradox; [71, 72]), the present study findings can be attributed to multiple factors, one of which includes the role of acculturation. For example, Abraido-Lanza and colleagues [73] found that more acculturated Latinos in the U.S. had a greater likelihood of high alcohol intake, current smoking, and high BMI, which are all risk factors for T2DM complications. On the other hand, there are a number of protective factors associated with maintaining aspects of one’s cultural beliefs, values, norms, and practices [35] that could help to explain the beneficial effects of foreign born status on HbA1c as noted in the present study.

The finding that social-structural, cultural, psychological, and self-care variables accounted for a notable proportion of the variance in the biological outcome HbA1c, has conceptual as well as clinical implications. While the Integrative Model of Culture, Psychology, and Health Behavior [23, 24, 35] has been employed to better understand the influence of social-structural, cultural, and psychological factors on health behaviors such as cancer screening, healthy eating, and physical activity [19, 40, 43], it has only recently been used as a conceptual framework for explaining biological outcomes (see [20, 74]). Results from the present study provide additional evidence concerning the mechanisms by which culture may impact biology, highlighting the critical role of psychological and behavioral phenomena. Namely, structural equation modeling revealed that the influence of culture on diabetes self-care was indirect, through the influence of diabetes distress. The present study draws attention to the importance of not only testing the effect of culture on behaviors and outcomes relevant to T2DM, but also the need to test for mediating psychological factors relevant to the cultural and behavioral phenomena of interest.

From a clinical perspective, the fact that the hypothesized model including social structural, cultural, psychological, and behavioral factors relevant to T2DM accounted for 13.2% of the variance in the biological outcome HbA1c, a marker of disease progression, is significant. Paddison and colleagues [75] conducted a study with T2DM patients in Australia and found that psychological perceptions of diabetes accounted for approximately 8% of differences in metabolic control. The authors argued that because these psychological factors made a unique and statistically significant impact on physiological outcomes, modification of psychological views could help to produce more positive disease outcomes. Based on findings from the present study, it could be argued that interventions which target both cultural and psychological factors could produce an even greater impact on T2DM outcomes. Still, due to the use of cross-sectional data in the present study, there is a possibility that individuals with poor glycemic control in turn experience greater diabetes distress and cultural beliefs about social exclusion. Although the structural equation model (SEM) tested in the present study was hypothesized a priori based on theory, and results from SEM did not support a path from HbA1c to diabetes distress or cultural beliefs, a more definitive test of these relations using longitudinal data is warranted.

The development of culturally relevant interventions designed to reduce diabetes-related distress among Latino patients with T2DM is clearly warranted. While research points to the success of interventions aimed at reducing diabetes distress, their impact on diabetes self-management is only modest [76]. At the same time, a systematic review revealed that culturally tailored lifestyle interventions for diabetes prevention were modestly effective in reducing the risk of diabetes among Latinos in the U.S. [77]. Interestingly, few of the interventions included in that systematic review specifically addressed cultural beliefs about diabetes. One might argue that in the case of interventions with diabetes patients, as compared to interventions aimed at preventing the onset of diabetes, it would be even more critical to address patients’ cultural beliefs about T2DM. Collectively, these intervention studies suggest that perhaps simultaneously addressing both cultural and psychological factors could produce more significant effects on diabetes self-care and outcome. Such an approach could contribute to reducing the noted disparities associated with T2DM complications and mortality rates between Latinos and NLW in the U.S.

Considering that the cultural belief of social rejection and exclusion is shared among Latino patients with T2DM and associated with heightened diabetes distress, interventions could benefit from addressing this particular cultural barrier while also drawing from potentially protective cultural aspects. For example, interventions could take into consideration familism, a cultural value considered central to some Latinos, which emphasizes warm, close, supportive family relationships and the prioritization of the family over the self [78, 79]. Research indicates that familism is associated with increased social support and better psychological health [80], and therefore may serve as a potential protective factor in reducing diabetes distress and risk for disease complications. This cultural value could be particularly beneficial in reducing Latino diabetes patients’ cultural beliefs about social exclusion and rejection due to their disease while at the same time reducing the likelihood of diabetes distress.

The current study also highlights opportunities for future research. For example, there is a clear need for research with other racial/ethnic minority populations (e.g., Black, Native American) and low SES populations that are disproportionally affected by T2DM and likely experience increased diabetes distress associated with poor self-care and outcomes. Hence, the cultural research approach and Integrative Model utilized in the present study could also be applied with other populations experiencing disparities in T2DM as well as within the context of other chronic diseases (e.g., hypertension, cardiovascular disease, and other metabolic syndromes) that pose significant public health concerns. In line with this, future research could especially benefit from employing an integrative approach to the study of culture on health behavior using robust statistical techniques (e.g., multi-group SEM) to investigate contributing cultural, psychological, and behavioral factors on specific chronic diseases, and identify and/or clarify pathways to poor chronic disease outcomes among socially, economically, or educationally disadvantaged populations.

In light of the study findings, there are some limitations of the research that should be considered. First, although this research includes a relatively large clinical population of Latino patients with T2DM, for the purposes of structural equation modeling this sample size may have resulted in greater difficulty to detect additional significant paths. Still, findings from the use of the Yuan-Bentler statistic for small sample sizes suggested excellent model fit [59] and our power analysis indicated that we were only two participants short of the suggested minimum sample size for detecting significant effects. Second, the biological outcome HbA1c was self-reported, which is not as preferable as obtaining this data from a chart review. Still, the diabetes treatment center conducted a test of participants’ HbA1c one week prior to data collection, suggesting that the self-report may be less vulnerable to memory bias. In a similar vein, the global item used to assess diabetes self-care, may also be vulnerable to subjectivity and self-report bias. Third, results are based on findings from a sample recruited exclusively from Southern California, with participants predominantly identifying as Mexican American. As such, it is unclear whether results from the current study would be the same with Latino patients from other regions of the U.S. that represent individuals from different Latin American origins. Moreover, participants were recruited at the second of four education classes at a diabetes treatment center. Hence, the generalizability of the study findings to other populations of T2DM patients such as those who could not attend education classes, were uninteresting in attending, or who only attended one class should be viewed with caution. Fourth, although all participants self-identified as Latin American, due to formatting issues with our instrument, over half did not further specify their specific ethnic background (e.g., Mexican American, Cuban American, etc.). Hence, it was not possible to examine within group differences in the study variables. Finally, while the tested propositions are solidly grounded in theory that operationalizes culture as a relatively stable construct, the use of a cross-sectional design limits the test of temporal relations. Therefore, future research utilizing a longitudinal methodological approach could be particularly beneficial to rule out the possibility of bidirectional relations [81].

Despite these limitations, this study has several notable strengths. The present study demonstrated the advantages of examining the role of culture in health behavior using an integrated framework by simultaneously testing the effects of social-structural, cultural, and psychological factors as determinants of diabetes self-care and outcome. Furthermore, latent variable statistical modeling techniques allowed for a comprehensive examination of these relationships simultaneously, rather than focusing on a test of these individual variables. This study also employed a previously validated cultural instrument developed using the mixed-methods cultural research approach to instrument development [23]. As such, it was possible to investigate a cultural variable considered central to Latino patients’ experiences of T2DM, thereby improving the translatability of the research findings necessary for the development of culturally relevant interventions. Finally, the current study is particularly significant from a public health perspective, given that the research was conducted in a region of Southern California with high rates of T2DM as well as some of the worst diabetes mortality rates [41].

## Footnotes

1 For the purpose of this article, Latin Americans (Latinos) in the U.S. refers to individuals who themselves or their ancestors are originally from Latin America or regions of the U.S. that used to be part of Latin America. This is different from the term Hispanic, which includes people from all Spanish-speaking countries [1].
